# The Clinical Impact of Change in the Neutrophil to Lymphocyte Ratio During the Perioperative Period in Gastric Cancer Patients Who Receive Curative Gastrectomy

**DOI:** 10.1007/s12029-023-00976-7

**Published:** 2023-10-27

**Authors:** Toru Aoyama, Itaru Hashimoto, Yukio Maezawa, Kentaro Hara, Keisuke Kazama, Masakatsu Numata, Kazuki Otani, Sho Sawazaki, Haruhiko Cho, Junya Morita, Shinnosuke Kawahara, Mie Tanabe, Norio Yukawa, Aya Saito, Takashi Ogata, Yasushi Rino, Takashi Oshima

**Affiliations:** 1 Department of Surgery, Yokohama City University, Yokohama, Japan; 2 Department of Gastrointestinal Surgery, Kanagawa Cancer Center, Yokohama, Japan; 3 Department of Surgery, Tokyo Metropolitan Cancer and Infectious Diseases Center Komagome Hospital, Tokyo, Japan

**Keywords:** Gastric cancer, Neutrophil, Lymphocyte, Survival

## Abstract

**Aim:**

Recently, change in the neutrophil to lymphocyte ratio (cNLR) has been shown to be a promising prognostic inflammation marker in some malignancies. The aim of the present study was to evaluate the clinical impact of the cNLR in gastric cancer patients who received curative gastrectomy.

**Patients and Methods:**

The present study included 450 patients who underwent curative treatment for gastric cancer between 2013 and 2017 at Kanagawa Cancer Center. The cNLR was defined as follows: cNLR = NLR at 1 month after surgery–NLR at 1 week before surgery. The prognosis and clinicopathological parameters of the increased cNLR and decreased cNLR groups were analyzed.

**Results:**

The OS stratified by each clinical factor was compared using the log-rank test, and a significant difference was observed using a cutoff value of cNLR at 0.762. When comparing the patient background factors between the increased cNLR (≥ 0.762) and decreased cNLR (< 0.762) groups, there were no significant differences in age, sex, T status, or N status. In the increased cNLR group, the OS rates at 3 and 5 years after surgery were 87.5% and 77.3%, respectively, while those in the decreased cNLR group were 92.8% and 87.3%, which amounted to a statistically significant difference (*p* = 0.041). The univariate and multivariate analyses of factors associated with OS showed that cNLR was a significant prognostic factor. When the postoperative course was compared between the two groups, the incidence rates of postoperative surgical complications and other-cause death were significantly higher in the increased cNLR group (*p* = 0.001 and *p* = 0.005, respectively).

**Conclusion:**

The cNLR is one of the significant risk factors in gastric cancer patients. Our results suggested that the changes of inflammation status during perioperative periods might be a promising prognostic factor for gastrointestinal cancer patients.

## Introduction

Gastric cancer is the fourth most common cancer and the second leading cause of cancer-related death in the world [1, 2]. The prognosis of gastric cancer is gradually improving with the improvement of minimally invasive surgery, perioperative care, and perioperative adjuvant treatment [3–5]. However, almost half of patients experience recurrence even after curative treatment [6, 7]. It is necessary to identify the prognostic factors and/or predictors of perioperative adjuvant treatment. To date, several prognostic factors and predictors have been evaluated in gastric cancer. Recently, the systemic inflammation status was shown to affect short- and long-term oncological outcomes [8]. Among them, the neutrophil to lymphocyte ratio (NLR) is a promising prognostic factor. The NLR is identified as a nonspecific marker of systemic inflammation. Some studies have shown that gastric cancer patients with a high NLR have a significantly poorer prognosis than those with a low NLR [9–11]. On the other hand, the inflammation status dramatically changes during the perioperative period. The optimal timing for the evaluation of the NLR has not been well evaluated in previous studies. Moreover, a one-point measurement of the NLR, such as in the pretreatment period or postoperative period, may not fully reflect the clinical significance. Considering these factors, we hypothesize that the change in the NLR during treatment would have a greater clinical impact than a one-point measurement in the management of patients undergoing gastric cancer treatment. To confirm our hypothesis, we evaluated the clinical impact of the change in the NLR during the perioperative period in gastric cancer patients who received curative treatment.

## Patients and Methods

### Patients

The medical records of consecutive esophageal cancer patients who underwent curative resection at Kanagawa Cancer Center from 2013 to 2017 were retrospectively reviewed. All patients had histological diagnosis of adenocarcinoma, were judged to have clinical stage I–III disease based on the 15th edition of the general rules for gastric cancer reported by the Japanese Gastric Cancer Association, received curative gastrectomy as a primary treatment for gastric cancer, and received complete resection (R0) of gastric cancer with radical lymph node dissection.

### Surgical Procedure and Adjuvant Treatment

In all cases, patients received gastrectomy with nodal dissection. D1 + nodal dissection and D2 dissection were performed for patients with clinical stage IA disease and clinical stage ≥ IB disease, respectively. Patients with pathological II or III disease were treated with S-1-based adjuvant chemotherapy within 6 weeks after surgery [12–15].

### Measurement of the NLR and Change in the NLR (cNLR)

The neutrophil lymphocyte ratio (NLR) was calculated from the absolute neutrophil count and the absolute lymphocyte count. The NLR was measured 1 week before surgery and 1 month after surgery. Changes in the NLR were defined as follows: cNLR = NLR at 1 month after surgery–NLR at 1 week before surgery.

### Follow-up

The patients were followed up at outpatient clinics. At least every 3 months for five years, the patients underwent hematological tests (including CEA and CA19-9 tumor marker level measurements) and physical examinations. In addition, every 3 months during the first 3 years after surgery and every 6 months until 5 years after surgery, the patients underwent computed tomography (CT) examinations.

### Evaluations and Statistical Analyses

Differences in the cNLR and clinicopathological parameters were analyzed by the chi-square test. Overall survival (OS) and recurrence-free survival (RFS) were analyzed by the Kaplan–Meier method. A Cox proportional hazards model was used to perform univariate and multivariate survival analyses. We used stepwise methods for multivariate analysis in the present study. The *p* value cutoff was set at *p* < 0.05. The SPSS software program (v27.0 J Win; IBM, Armonk, NY, USA) was used for all statistical analyses. This study was approved by the IRB of Kanagawa Cancer Center.

## Results

### Patients

Four hundred fifty gastric cancer patients were included in the present study. Among them, 301 patients (66.9%) were male, and 315 patients (70.0%) were > 65 years of age. Before treatment, the median neutrophil count was 3270 (range 1110–9280), the median lymphocyte count was 1630 (range 0–4753), and the median NLR was 1.995 (range 0–14.6). At 1 month after surgery, the median neutrophil count was 3170 (range 730–9750), the median lymphocyte count was 1633 (range 0–4930), and the median NLR was 1.934 (range 0.40–28.5).

### Survival Analysis

**Fig. 1:**
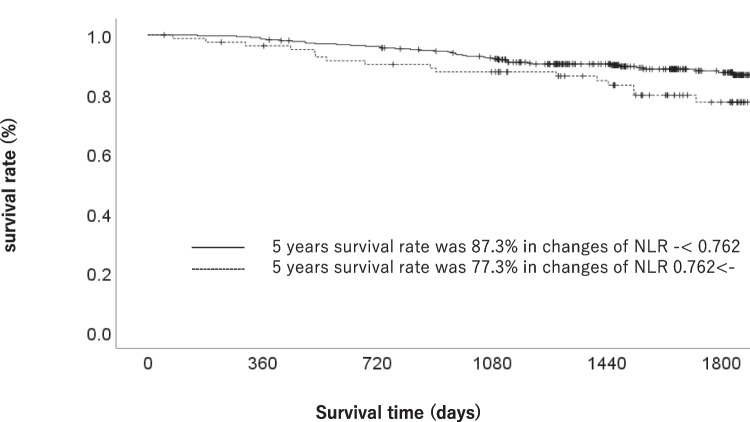
The overall survival of gastric cancer patients in the increased cNLR group (≥ 0.762) and the decreased cNLR group (< 0.762)

**Fig. 2:**
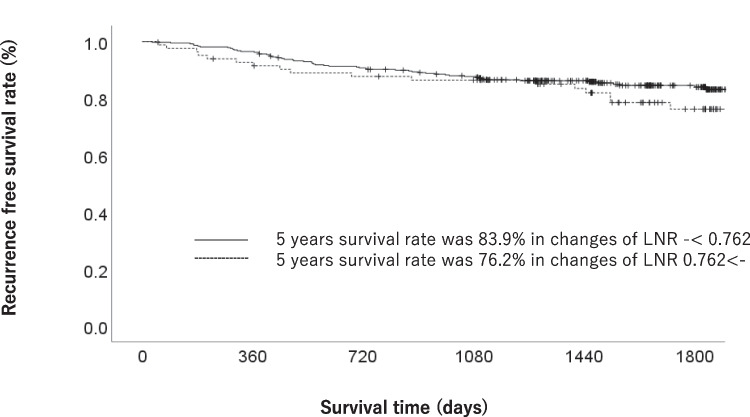
Recurrence-free survival of gastric cancer patients in the increased cNLR group (≥ 0.762) and the increased cNLR group (< 0.762)

The OS stratified by each clinical factor was compared using a log-rank test, and a significant difference was observed using a cutoff value of cNLR 0.762 (Table 1). When comparing the patient backgrounds between the increased cNLR (≥ cNLR 0.762) and decreased cNLR (cNLR < 0.762) groups, patient backgrounds factors, such as median age, sex, T factor, and N factor, were similar. Each clinicopathological factor was categorized as shown in Table 2 and analyzed for its prognostic significance. The univariate and multivariate analyses of factors associated with OS showed that pathological N factor, vascular invasion, and cNLR were significant prognostic factors. The cNLR was therefore selected for the final multivariate analysis model. In the increased cNLR group, the OS rates at 3 and 5 years after surgery were 87.5% and 77.3%, respectively, while those in the decreased cNLR group were 92.8% and 87.3%, which amounted to a significant difference (*p* = 0.041). The OS curves are shown in Fig. 1. The RFS rates of the increased cNLR and decreased cNLR groups were similar. In the increased cNLR group, the RFS rates at 3 and 5 years after surgery were 86.4% and 76.2%, respectively, while those in the decreased cNLR group were 87.6% and 83.6%. cNLR was not selected for the final multivariate analysis model for RFS (Table 3). The RFS curves are shown in Fig. 2.

**Table 1:** Patient characteristics

Characteristics	No. of patients (%)	1-year OS rate (%)	3-year OS rate (%)	5-year OS rate (%)	*p* value
Age (years)					0.049
< 65	135 (30%)	99.3	97.0	91.1	
≥ 65	315 (70%)	97.8	89.3	82.9	
Gender					0.032
Man	301 (66.9%)	98.0	89.9	82.8	
Woman	149 (33.1%)	98.6	95.2	90.5	
Pathological type					0.391
Intestinal	213 (47.3%)	98.6	93.4	86.6	
Diffuse	237 (52.7%)	97.9	90.0	84.3	
UICC T status					< 0.001
T1	277 (61.6%)	99.3	96.3	92.5	
T2 to T4	173 (38.4%)	96.5	84.1	73.8	
Lymph node metastasis					< 0.001
Negative	324 (72.0%)	98.8	96.3	91.9	
Positive	126 (28.0%)	96.8	79.7	67.8	
Lymphatic invasion					< 0.001
Negative	310 (68.9%)	98.4	95.1	91.4	
Positive	140 (31.1%)	97.8	83.9	71.5	
Vascular invasion					< 0.001
Negative	255 (56.7%)	99.2	97.6	93.5	
Positive	195 (43.3%)	96.9	83.9	74.5	
Change of NLR					0.046
- < 0.762	368 (81.7%)	98.6	92.8	87.3	
0.762 ≤ -	82 (18.3%)	96.3	87.5	77.3	
Postoperative complications					0.571
Yes	65 (14.4%)	96.9	90.6	82.4	
No	385 (85.6%)	98.4	91.8	85.9	

*OS* overall survival, *UICC* Union for International Cancer Control, *NLR* neutrophil–lymphocyte ratio

**Table 2:** Uni- and multivariate Cox proportional hazards analysis of clinicopathological factors for overall survival

Factors	No	Univariate analysis			Multivariate analysis		
		OR	95%CI	*p* value	OR	95%CI	*p* value
Age (years)				0.053			
- < 65	135	1.000					
65≦-	315	1.911	0.991–3.685				
Gender				0.035			
Woman	149	1.000					
Man	301	1.978	1.048–3.733				
Pathological type				0.392			
Intestinal	213	1.000					
Diffuse	237	1.255	0.746–2.111				
UICC T status				< 0.001			
T1	277	1.000					
T2–T4	173	4.035	2.312–7.040				
Lymph node metastasis				< 0.001			< 0.001
Negative	324	1.000			1.000		
Positive	126	4.619	2.726–7.826		3.169	1.812–5.544	
Change of NLR				0.049			0.041
- < 0.762	368	1.000			1.000		
0.762 < -	82	1.783	1.002–3.171		1.828	1.026–3.255	
Lymphatic invasion				< 0.001			
Negative	310	1.000					
Positive	140	3.777	2.230–6.398				
Vascular invasion				< 0.001			< 0.001
Negative	255	1.000			1.000		
Positive	195	4.773	2.613–8.718		3.176	1.677–6.015	
Postoperative complications				0.572			
No	385	1.000					
Yes	65	1.217	0.616–2.407				

*UICC* Union for International Cancer Control, *NLR* neutrophil–lymphocyte ratio

**Table 3:** Uni- and multivariate Cox proportional hazards analysis of clinicopathological factors for recurrence-free survival

Factors	No	Univariate analysis			Multivariate analysis		
		OR	95%CI	*p* value	OR	95%CI	*p* value
Age (years)				0.060			
- < 65	135	1.000					
65≦-	315	1.723	0.977–3.040				
Gender				0.242			
Woman	149	1.000					
Man	301	1.359	0.812–2.273				
Pathological type				0.284			
Intestinal	213	1.000					
Diffuse	237	1.289	0.810–2.050				
UICC T status				< 0.001			
T1	277	1.000					
T2–T4	173	3.357	2.078–5.422				
Lymph node metastasis				< 0.001			< 0.001
Negative	324	1.000			1.000		
Positive	126	4.564	2.860–7.283		2.599	1.501–4.499	
Change of NLR				0.218			
- < 0.762	368	1.000					
0.762 ≤ -	82	1.406	0.817–2.420				
Lymphatic invasion				< 0.001			0.026
Negative	310	1.000			1.000		
Positive	140	3.945	2.467–6.308		1.892	1.079–3.317	
Vascular invasion				< 0.001			0.022
Negative	255	1.000			1.000		
Positive	195	3.526	2.137–5.818		1.926	1.099–3.374	
Postoperative complications				0.214			
No	385	1.000					
Yes	65	1.447	0.808–2.593				

*UICC* Union for International Cancer Control, *NLR* neutrophil–lymphocyte ratio

### Postoperative Course of the Low- and High-NLR Groups

When the postoperative course of the two groups was compared, there were some differences in the incidence of postoperative surgical complications and the incidence of other-cause death. The incidence of postoperative complications was 11.7% (43/368) in the decreased cNLR group and 26.8% (22/82) in the increased cNLR group. The incidence of surgical complications was significantly higher in the increased cNLR group than in the decreased cNLR group (*p* = 0.001). The incidence rates of other-cause death were 3.5% (13/368) in the decreased cNLR group and 11.0% (9/82) in the increased cNLR group, which amounted to a statistically significant difference (*p* = 0.005). On the other hand, there was no significant difference in the site of first relapse at lymph node metastasis between the two groups (Table 4).

**Table 4:** Patterns of recurrence between the patients with NLR ‐ < 0.05 and those with NLR 0.05 ≤ ‐

			NLR ratio				
	All cases (*n* = 450)		- < 0.762 (*n* = 368)		0.762 <—(*n* = 81)		
Recurrence site	Number	%	Number	%	Number	%	*p* value
Peritoneal	17	3.8%	16	4.3%	1	1.2%	0.179
Hematological	13	2.9%	10	2.7%	3	3.7%	0.645
Lymph node	16	3.6%	12	3.3%	4	4.9%	0.475
Local site	4	0.9%	4	1.1%	0	0.0%	0.343

*NLR* neutrophil–lymphocyte ratio

## Discussion

The aim of the present study was to clarify the clinical impact of change in the NLR (cNLR) in gastric cancer patients who received curative treatment. The major finding is that the cNLR is a significant risk factor in gastric cancer patients. In addition, an increased cNLR is related to postoperative complications and other-cause death. Therefore, the cNLR is a promising prognostic factor for gastric cancer patients.

In the present study, an increased cNLR was associated with a significantly poorer prognosis in comparison to a decreased cNLR (hazard ratio 1.828, 95% confidence interval 1.026–3.255, *p* = 0.041). Moreover, the 5-year OS rate of patients with a decreased cNLR was 87.3%, while that of patients with an increased cNLR was 77.3%. Although limited studies have evaluated the clinical impact of the cNLR in malignancies, similar results were observed in previous studies. Wang et al. evaluated the change and prognostic impact of the NLR during chemotherapy in 120 patients with unresectable gastric cancer [16]. They found that an increased cNLR during chemotherapy was significantly associated with shorter OS. The median OS was 9.0 months in patients with an increased cNLR and 20 months in those with a decreased cNLR (*p* < 0.001). Min et al. evaluated the change in the NLR during neoadjuvant chemotherapy in 111 advanced gastric cancer patients who received neoadjuvant chemotherapy and curative surgery [17]. They found that patients with a decreased NLR after neoadjuvant chemotherapy showed significantly longer OS (*p* < 0.001) and DFS (*p* < 0.001) in comparison to patients with an increased NLR. In addition, they demonstrated that the hazard ratio of change in the NLR for OS was 1.945 (95% CI 2.180–22.438, *p* = 0.001), while that for disease-free survival was 1.753 (95% CI 1.861–17.900, *p* = 0.002). Considering the results of the present study and previous reports, change in the LNR during cancer treatment might be a promising prognostic factor.

Why does a change in NLR affect patient survival? There are some possible explanations for this issue. First, change in the NLR was associated with the occurrence of postoperative complications. Recent studies have shown that the occurrence of postoperative complications affects long-term oncological outcomes in various malignancies [18–20]. Therefore, the prognosis might have worsened in patients with an increased NLR due to postoperative complications. Second, change in the NLR might affect the perioperative adjuvant treatment response. Hoshino et al. evaluated the clinical impact of change in the NLR during neoadjuvant chemotherapy in 209 esophageal cancer patients who underwent neoadjuvant chemotherapy and curative surgery [21]. They demonstrated that the NLR reduction group had a significantly higher RFS than the non-NLR reduction group (3-year RFS, former 70.3% vs. latter 41.2%; *p* = 0.012). Moreover, they clarified the clinical relationship between change in the NLR and the neoadjuvant chemotherapy response. They demonstrated that a change in the NLR was significantly associated with the histological response (odds ratio 3.80, 95% CI 1.69–8.58, *p* = 0.001). Although the present study did not evaluate the perioperative adjuvant treatment response, 25% of the patients in the present study received adjuvant treatment. Changes in the NLR might affect the response to and/or feasibility of adjuvant treatment in these patients. However, the precise mechanism is unclear. Further studies should be conducted to focus on this issue.

To introduce change in the NLR in daily clinical practice, it is necessary to set the optimal cutoff value for change in the NLR. In the present study, we set the cutoff value of change in NLR as 0.762 according to the 1-, 3-, and 5-year survival rates. Previously, several cutoff values of change in NLR have been reported. Wang et al. set the cutoff value at 4.62, while Wang et al. set it at 1.75 [16, 17]. These differences might be due to the following reasons. First, the number of patients and background factors were different. Our study (*n* = 450) evaluated patients with resectable gastric cancer, including stage I to III disease, Wang et al. evaluated unresectable gastric cancer (*n* = 120), and Liu et al. evaluated advanced gastric cancer (*n* = 111). Second, the methods used to evaluate change in the NLR were different. Our study evaluated the cutoff value of change in the NLR according to the patient’s survival rate, while other studies evaluated the cutoff value of change in the NLR according to receiver operating characteristic curves. One of the best ways to detect the cutoff value of cNLR is using ROC curve. We used ROC curve to detect the cutoff value of cNLR in the present study (data not shown). However, we did not detect the optimal cutoff value of cNLR. Instead, we determined the cutoff value considering the point which separates the survival. The 5-year survival rate was clearly separated by cNLR at 0.762. Therefore, we set the cutoff value at 0.762. Our study suggested that increased NLR during perioperative periods seemed to have poor prognosis. The appropriate cutoff value should be determined in the other validation studies.

The present study was associated with some limitations. First, this was a retrospective study with a relatively small sample size from a single institution. Thus, there may have been a selection bias. Second, there may have been a time bias in the present study. Our study included data from 2012 to 2017. During this period, perioperative care and perioperative adjuvant treatment were improved. Third, in the present study, the incidence of other causes of death was significantly higher in the increased cNLR group than in the decreased cNLR group. However, the precise mechanism underlying this issue is unclear. Considering these factors, our results need to be validated in another large cohort.

In conclusion, the cNLR is one of the significant risk factors in gastric cancer patients. Our results suggested that the changes of inflammation status during perioperative periods might be a promising prognostic factor for gastrointestinal cancer patients.

## Data Availability

The anonymized data used and/or analyzed during the current study are available from the corresponding author upon reasonable request.
